# Supplementary material for: Pathophysiological Significance of Neutrophilic Transfer RNA-Derived Small RNAs in Asymptomatic Moyamoya Disease
Source: Cells. 2021 May 1;10(5):1086. doi: 10.3390/cells10051086 (PMC8147334; doi:10.3390/cells10051086)

| 2D Structure | Local AU | Position |
|---|---|---|

```
3710                              3740
                           7mer-m8
5'-agtAATCTGGATGAATGAATAAAATGTGGGg-3' utr
      ||||: :|:    |   |   |||||||
3'-uccUUAGGAUUGGCGA--UCUGGUACACCCu-5' tRF
               161514  13    7 6 5 4 3 2
              3' Pairing      Seed
```

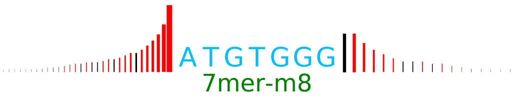


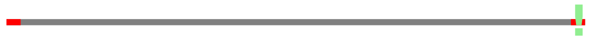

Supplement: Supplementary file 1 [file cells-10-01086-s001.zip › cells-1149161 3rd supplementary materials/Data Supplements/Data Supplement V.zip!/Data supplement 6-mRNAs targets of tsRNAs/tsRNA-Glu-TTC-010/Diagrams/tRF-Glu-TTC-010_vs_ENST00000300571(GPRC5B).pdf]
